# Effect of Presenilin Mutations on APP Cleavage; Insights into the Pathogenesis of FAD

**DOI:** 10.3389/fnagi.2016.00051

**Published:** 2016-03-11

**Authors:** Nuomin Li, Kefu Liu, Yunjie Qiu, Zehui Ren, Rongji Dai, Yulin Deng, Hong Qing

**Affiliations:** School of Life Science, Beijing Institute of TechnologyBeijing, China

**Keywords:** Familial Alzheimer’s disease, γ-secretase, presenilin1, amyloid β-peptide

## Abstract

Alzheimer disease (AD) is characterized by progressive memory loss, reduction in cognitive functions, and damage to the brain. The β-amyloid precursor protein can be sequentially cleaved by β- secretase and γ-secretase. Mutations in the presenilin1(PS1) are the most common cause of Familial Alzheimer’s disease (FAD). PS1 mutations can alter the activity of γ-secretase on the cleavage of the β-amyloid precursor protein, causing increased Aβ production. Previous studies show that the βAPP-C-terminal fragment is first cleaved by β-scretase, primarily generating long fragments of Aβ48 and Aβ49, followed by the stepwise cleavage of every three amino acid residues at the C terminus, resulting in Aβ48-, 45-, 42 line and Aβ49-, 46-, 43-, 40 line. Here, we used LC-MS/MS to analyze unique peptides IAT, VVIA, ITL, TVI, IVI through sequential cleavage, combined with ELISA to test the level of Aβ42 and Aβ40 for validation. The results show that most FAD mutant PS1 can alter the level of Aβ42 and Aβ40 monitored by the Aβ42/Aβ40 ratio. Among them, six mutants (I143T, H163P, S170F, Q223R, M233V, and G384A) affect the Aβ42/40 ratio through both Aβ49-40 and Aβ48-38 lines; L166P through decreasing the Aβ49-40 line, six mutants (I143V, M146V, G217A, E280A, L381V, and L392V) through increasing the Aβ48-42 line. More importantly, we found some mutations can affect the γ-secretase cleavage preference of α-CTF and β-CTF. In conclusion, we found that the FAD PS1 mutations mainly increase the generation of Aβ42 by decreasing the cleavage of Aβ42–Aβ38 and Aβ43–Aβ40.

## Introduction

Alzheimer disease (AD) is the most common neurodegenerative disease. One of the main etiological hallmarks of AD is excessive production of Aβ (Bentahir et al., 2006; Cacquevel et al., 2012). Aβ peptides aggregate and deposit into soluble oligomers, fibrils, and senile plaques, which are closely associated with synaptic dysfunction and neuronal network perturbations, finally causing gross atrophy of the brain (Lesne et al., 2006; Shankar et al., 2008; Sun et al., 2015). Aβ is a 38∼43 amino acid peptide derived from the β-APP through sequential cleavage by β-secretase (BACE1) and γ-secretase (Adlard et al., 2008; Thinakaran and Koo, 2008; Barnwell et al., 2014). BACE1 is an aspartyl protease β-site APP cleaving enzyme1 that cleaves APP mainly at a unique site, whereas the γ-secretase complex cleaves the CTF at several sites, with preference for positions 40 and 42, forming the Aβ_1-40_ and Aβ_1-42_ peptides (Jonsson et al., 2012). Previous studies indicated that compared to other forms, such as Aβ_1-4_, Aβ_1-38_, Aβ_1-42_, and Aβ_3-40_ are more amyloidogenic (Chow et al., 2010). In particular, Aβ42, which is more prone to aggregate and form soluble oligomers that eventually form insoluble plaques, is more amyloidogenic (Kakuda et al., 2006; Czirr et al., 2008). Clinical studies indicated that Aβ_1-42_ peptide showed a higher percentage concentration in AD patients (Burdick et al., 1992; Kim et al., 2007). In the physiological condition, more than 90% of Aβ is a shorter form of Aβ40 and less than 5% of Aβ is a longer form of Aβ42 (Sun et al., 2015).

Autosomal dominant FAD is a rare form of AD and usually presents before the age of 65 years in individuals with a positive family history in at least three generations (Wu et al., 2012). Currently, more than 185 mutations have been identified in PS1, 13 in presenilin 2 (PS2) and 33 in APP among FDA patients (Cacquevel et al., 2012). PS1 is a *trans*-membrane protein that is an important component of the catalytic core of γ-secretase (Fernandez et al., 2014). γ-secretase, which is a multiprotein complex, is an unusual intramembranous cleaving aspartyl protease composed of presenilin, Nicastrin, Pen-2 and Aph-1 (Yu et al., 2000; Francis et al., 2002; Goutte et al., 2002; Christensen et al., 2004). PS1 is a highly conservative membrane protein, with nine TMDs. Large numbers of pathogenic mutations have been found throughout the coding sequence of PS1. Most of the PS1 mutations in FAD are located at TMDs (Wanngren et al., 2014), causing an increase in the Aβ42/Aβ40 ratio, either by decreasing the production of Aβ40 or increasing the production of Aβ42 (Bentahir et al., 2006; Shen and Kelleher, 2007; Kretner et al., 2011). Other PS1 mutations in FAD, such as mutations at D257A and D385A are dominant negative, can lead to decreased Aβ peptide secretion and the accumulation of the C-terminal fragments of the precursor protein (Kim et al., 2001). Previous studies showed that through γ-secretase, βAPP-C-terminal fragment (β-CTF) is cleaved at the 𝜀-site, generating primarily long fragments. Meanwhile, Aβ48 and Aβ49 is followed by stepwise cleavage of every three amino acid residues at the C terminus (Qi-Takahara et al., 2005). These findings led to the hypothesis that there are two Aβ product lines: Aβ40 and Aβ42. In this hypothesis, the Aβ40 product line represents the amino-terminal APP intracellular domain (AICD) 50–99 and Aβ49, Aβ46, Aβ43, Aβ40, and, the Aβ42 product line represents AICD 49–99 and Aβ48, Aβ45, Aβ42, Aβ38 (He et al., 2010).

The PS1 gene has been widely studied since the discovery of FAD. While initial studies indicated the role of PS1 mutations in increased Aβ42 production in FAD, it has now become clear that a series of pathogenic mutations caused impairments in other PS activities as well, such as Aβ40, AICD, NICD and so on. Some researchers even proposed that pathogenic mutations in PS might play a role in the impaired γ-secretase-dependent and γ-secretase-independent activities through a dominant-negative mechanism. However, the molecular mechanisms remain elusive for FAD. Both D257A NTF and D385A CTF have been shown to abolish the γ-secretase activity in wild type or pathogenic PS1 mutants (Kim et al., 2005). Other mutations have also been shown to affect the Aβ42/Aβ40 ratio: eight mutants (I143T, E280A, P284L, Δexon9, G384A, F386S, S390I, L392P) were found to increase Aβ42, eleven (I143T, L166P, A246E, L250S, E280A, P284L, Δexon9, P377M, G384A, L392V) decrease Aβ40, and twelve (I143T, L166P, A246E, E280A, P284L, Δexon9, R377M, G384A, F386S, S390I, L392P, L392V) decrease Aβ38. These results show that decreased Aβ38 and Aβ40 and increased Aβ42 production are common phenotypes of PS1 mutations in FAD. Still more mutations were reported, but their exact role in FAD are still unclear (Houlden et al., 2001; Piccini et al., 2007; Uttner et al., 2010; Kim et al., 2012).

Given the important role the Aβ42/Aβ40 ratio played in AD, it is very important to understand the mechanism that leads to this change for the study of amyloidosis processing and AD onset (Kim et al., 2007). Here, we studied 13 different FAD PS1 mutations, plus one dominant negative mutation that affects the production of Aβ through APP processing. We also quantitatively analyzed the triple and tetra peptide produced by two distinct lines of long amyloid β cleavage processes with mass spectrometry. Our studies indicate that different mutations affect the Aβ42/Aβ40 ratio through different mechanisms. Some decrease the cleavage of Aβ42 to 38 (VVIA), while others decrease the cleavage of Aβ43 to Aβ40 (IAT). Such results can help us to better understand the underlying mechanism of PS mutations during the onset of AD.

## Materials and Methods

### DNA Constructs and Mutagenesis

BACE1-myc-his, pcC99, pcC83, wild-type PS1 human cDNAs (PS1-WT) were obtained from Weihong Song lab (University of British Columbia, Vancouver, BC, Canada). Mutations in PSEN1, (namely, I143T, I143V, M146V, H163P, L166P, S170F, G217A, M233V, Q223R, E280A, L381V, G384A, D385A, and L392V) were generated by overlap extension PCR on the plasmid pcDNA4.1/PS1-WT using corresponding primers (Supplementary Table S1). The PCR fragments were then digested using EcoRI /HindIII, and subcloned into pcDNA4.1.

### Cell Culture and Transfection

Human embryonic kidney 293 (HEK 293) cells, stably expressing “Swedish” mtAPP695 and BACE1 (2EB2 cell line), were cultured in Dulbecco’s Modified Eagle Media, which is a Nutrient Mixture F-12 (GIBCO, CA) supplemented with 10% fetal bovine serum (FBS; GIBCO) and 1% penicillin/streptomycin (GIBCO). Stable cell lines were selected using 200 cug/ml Zeocin and G418 (Invitrogen). Human embryonic kidney 293 (HEK 293) cells were cultured in Dulbecco’s Modified Eagle Media (GIBCO, CA) supplemented with 10% FBS (GIBCO) and 1% penicillin/streptomycin (GIBCO). The cDNA constructs were transiently transfected into the cells using the Lipofectamine 2000 reagent (Invitrogen), according to the manufacturer’s instructions.

### Extraction of Tri-, Tetra-, and Pentapeptides from Living Cultured Cells

2EB2 cells were transfected with PS1 or PS1 with various mutations. HEK293 cells co-transfected with pcC99 or pcC83 and PS1 or PS1 with various mutations were cultured to confluence in 10 cm dishes. Protease inhibitors (Protease inhibitor Cocktail Tablets, Roche, 04693132001) and 1 mM 4-(2-Aminoethyl) benzenesulfonyl fluoride hydrochloride (AEBSF, sigma, A8456) were added into the conditioned medium 44 h after transfection. The cells were washed rapidly with ice-cold PBS, and then, immediately boiled for 2 min. The boiled samples were sonicated for 3 min and centrifuged. The supernatant was then concentrated using a speed vacuum concentrator, and finally, subjected to an LC-MS/MS analysis of the tripeptide and tetrapeptide (Okochi et al., 2013).

### Aβ ELISA

The conditioned medium was collected for an Aβ (Aβ40 and Aβ42, Invitrogen) level assay. Protease inhibitor and 4-(2-Aminoethyl) benzenesulfonyl fluoride hydrochloride were added to the conditioned medium 4 h before collection to prevent degradation of Aβ. The levels of Aβ species (Aβ40 and Aβ42) were measured by ELISA Kits according to the manufacturer’s instructions.

### Identification and Quantification of Cleavage Peptides by LC-MS/MS

An electrospray ionization tandem quadruple mass spectrometer, (Agilent 6460, USA) accompanied by ultra-performance liquid chromatography (Agilent 1260), was used to identify and quantify the cleavage peptides. Samples were maintained at 4°C in the auto sampler. To quantify each peptide, a combination of precursor ion product ion pair was monitored using multiple reaction monitoring (MRM) modes. MRM methods were measured by LC-MS/MS as described previously (Takami et al., 2009). The m/z values for these peptides were as follows: 502.7 and 199.2 for VVIAT; 425.7 and 261.2 for FLF; 345.8 and 215.1 for ITL; 329.8 and 185.2 for VIV; 303.7 and 185 for IAT; 331.8 and 185.1 for VIT; 331.8 and 173.1 for TVI; 401.2 and 171.1 for VVIA.

### Immunoblotting

Cells were lysed 48 h after transfection in a RIPA Lysis Buffer with 50 mM Tris-HCl (pH 7.4), 150 mM NaCl, 1% NP-40, 0.1% SDS, Protease Inhibitor cocktail (Roche) and AEBSF. Sonication was done using an Ultrasonic Cell Disruptor (Sonics). The lysates were centrifuged at 14000 *g* for 10 min at 4°C. The protein levels were determined by the Quick Start^TM^ Bradford protein assay (Bio-Rad, 500-0201). Cell lysate was subjected to SDS polyacrylamide gel electrophoresis (SDS-PAGE) with 16% Tris-tricine (Li et al., 2006; Schagger, 2006) or 10% Tris-glycine gels. The samples were then transferred to PVDF membranes (Millipore, 0.22 μm). For protein detection, membranes were blocked for 1 h with 5% milk, as well as incubated with the polyclonal C20 antibody against the last 20 C-terminus of human APP. The blots were developed using an ECL system and intensities of the bands were quantified with Image Lab^TM^ Software (Bio-Rad).

### Statistical Analysis

Quantifications were done from data generated during three independent experiments. Values represent mean ± standard error of the mean. Comparisons of more than two groups were carried out using one-way ANOVA and Dunnett’s *post hoc* test using PS1 WT values as the control group (Fernandez et al., 2014). Statistical significance between the two groups was determined by an unpaired two-tailed *t*-test. *P* < 0.05 was considered to be statistically significant.

## Results

### Human FAD PS1 Mutations Increase the Production of CTF

**FIGURE 1:**
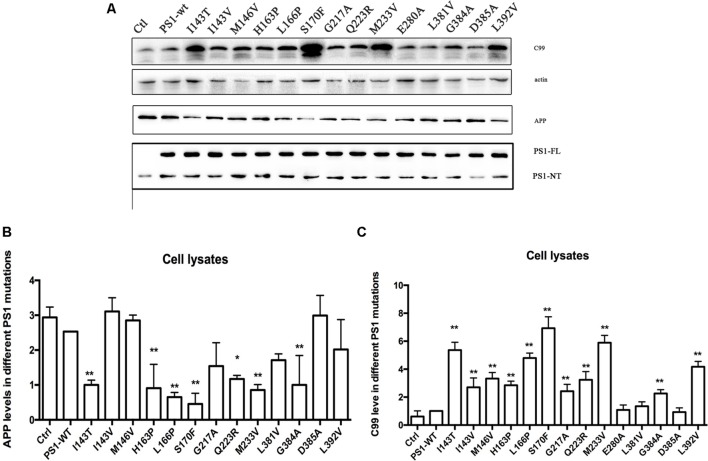
**Familial Alzheimer’s disease PS1 mutations affect CTF level.** Expression levels of APP, CTF in 2EB2 cells expressing empty vector, PS1 WT, I143T, I143V, M146V, H163P, L166P, S170F, G217A, Q223R, M233V, E280A, L381V, G384A, D385A, L392V. **(A)** Cell lysates from human Swedish mutant APP695 and BACE1 double expression stable cell line 2EB2 were analyzed by Western Blot. APP, APP CTFs were detected with the C20 polyclonal antibody, PS1-NT was detected with the rat anti-presenilin-1 monoclonal antibody (Millipore, MAB1563). **(B)** Quantification of APP in 2EB2 cells in **(A)**. **(C)** Quantification of C99 in 2EB2 cells in **(A)**. ^∗^*P* < 0.05; ^∗∗^*P* < 0.01.

In order to better understand the mechanisms of FAD PS1 mutations, 14 different PS1 mutations were selected and transiently transfected into 2EB2 cells that stably overexpressed Swedish APP and BACE1 (Qing et al., 2004). PS1 has nine TMDs and harbors the catalytic site with two conserved aspartate residues located in TMD6 and TMD7 (Wolfe et al., 1999; Henricson et al., 2005; Spasic et al., 2006). It was reported that most PS1 mutations are located in TMD2, TMD3, TMD4, TMD5, TMD6, and TMD7. Among them, TMD1-6 and TMD8-9 are hydrophobic. TMD7 has a partial hydrophilic catalytic cavity and is very sensitive to mutations, which dramatically reduce its capability to insert into the cell membrane (Wanngren et al., 2014). TMD7 is part of the hydrophilic catalytic cavity, which is inserted in the hydrophobic core of the membrane, and probably protected by stable hydrophobic domains that include TMD1-6 and TMD8-9. TMD6 is also susceptible to changes in amino acid residues. Fourteen different FAD PS1 mutations that we selected are located in TMDs. Four are located in TMD2 (I143T/V, M146V, H163P); one in TMD3 (L166P, S170F); one in TMD4 (G217A), two in TMD5 (Q223R, M233V); one in H7 (E280A); and, three in TMD7 (L381V, L392V, G384A). One mutant (D385A) was also used as a negative control (Uttner et al., 2010). Except for L381V and G384A that have been well-studied, the rest have rarely, if ever, before been studied. Vector alone, PS1 WT (wild type) or PS1 mutants, were transiently transfected into 2EB2 cells. Forty-eight hours after transfection, cells were collected, lysed, and a Western Blot was used to analyze the expression level of APP and CTFs, especially CTF99 expression. The levels of PS1 expression were used to verify the transfection efficiency. Compared with the vector alone (pcDNA4.1), both WT PS1 and PS1 with mutations could be transfected with high efficiency into the 2EB2 cell line. While the negative control mutation (D385A) did not affect the level of APP CTF expression, others, especially I143T, S170F, M233V, and L392V, increased the expression of APP CTF (**Figures 1A–C**).

### Human FAD PS1 Mutations Influence APP Cleavage Process

**FIGURE 2:**
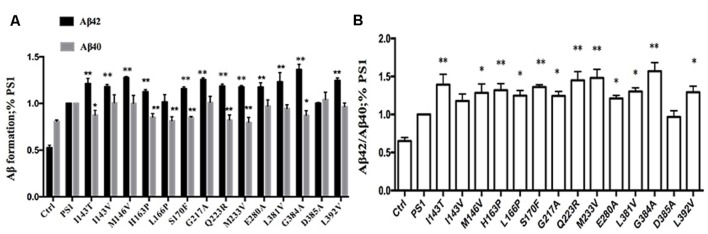
**Effects of PS1 WT and PS1 Mutants on long Aβ Cleavage in 2EB2 cell line by ELISA (A,B).** Conditioned medium from 2EB2 cells expressing empty vector, PS1-WT or FAD PS1 mutants were analyzed for levels of secreted Aβ40 and Aβ42 using ELISA kit. **(A)** The levels of Aβ40 and Aβ42 are plotted in relative to PS1 WT. **(B)** The Aβ42/Aβ40 ratio. ^∗^*P* < 0.05; ^∗∗^*P* < 0.01.

**FIGURE 3:**
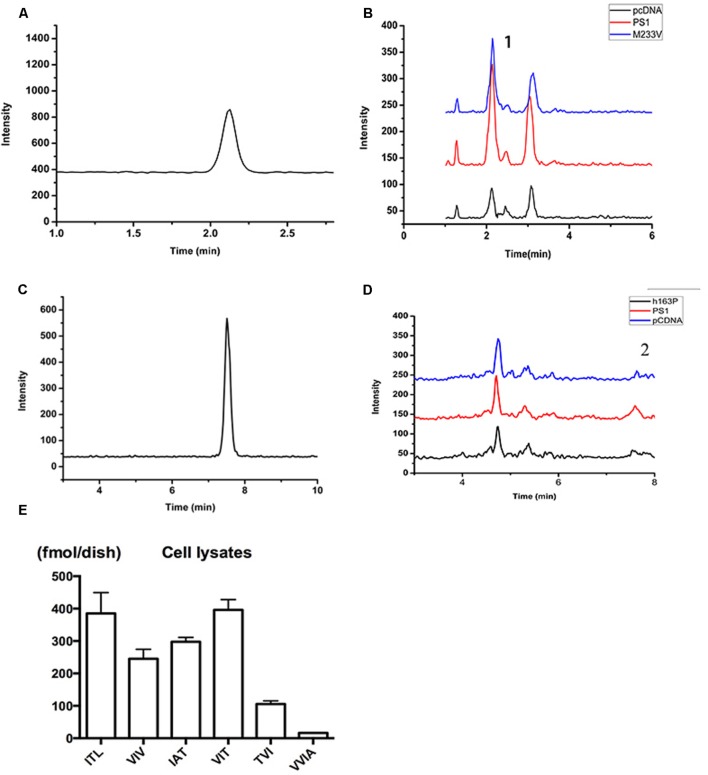
**Detection of related small peptide species of Aβ. (A)** The chromatographys of IAT standard peptides. **(B)** The Intensity of multiple reactions monitoring (MRM) ion chromatography of ITA. (1) Its transition is m/z = 303.7/185. Black, pCDNA4.1; Red, PS1; Blue, M233V. **(C)** The chromatographys of VVIA standard peptides. **(D)** The Intensity of multiple reactions monitoring (MRM) ion chromatography of VVIA. (2) Its transition is m/z = 401.2/171.1. Black, pCDNA4.1; Red, PS1; Blue, M233V. **(E)** Aβ species in lysates of 2EB2 cells in a 10 cm dish overexpressing PS1 WT.

**FIGURE 4:**
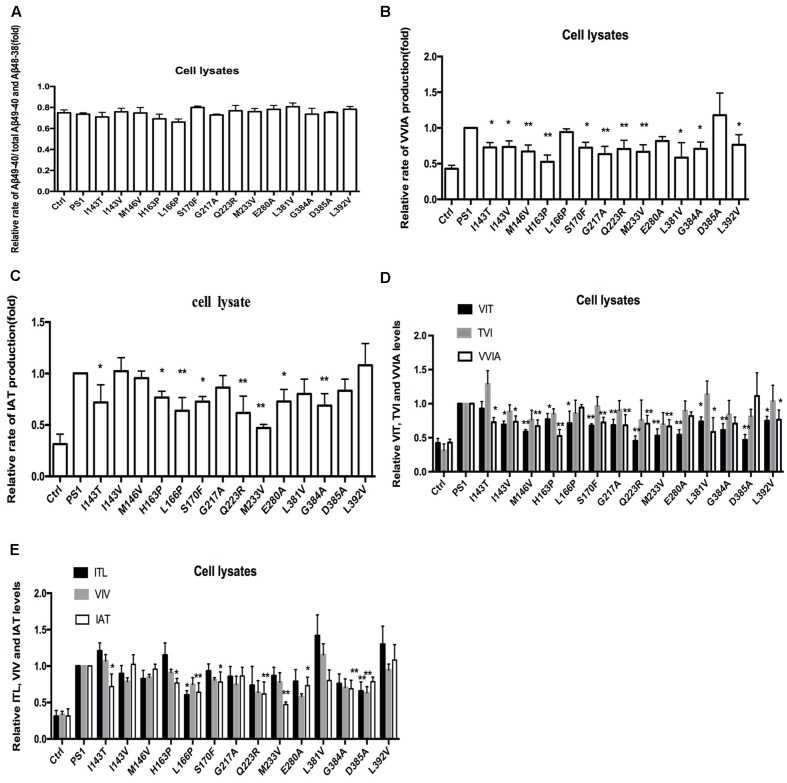
**Effects of PS-WT1 and PS1 Mutants on long Aβ Cleavage in 2EB2 cell line by LC-MS/MS. (A)** Fold changes of the relative rate of Aβ49-40/total Aβ production. **(B)** Fold changes of the relative VVIA levels in cell lysates of 2EB2 cell line. **(C)** Fold changes of the relative IAT levels in cell lysates of 2EB2 cells line. **(D)** Fold changes of the relative VIT, TVI, and VVIA levels in cell line expressing PS1 and PS1 FAD mutants. **(E)** Fold changes of the relative ITL, VIV, and IAT levels in cell line expressing PS1 and PS1 FAD mutants. ^∗^*P* < 0.05; ^∗∗^*P* < 0.01.

To investigate the effect of human FAD PS1 on the APP cleavage process, empty vector (pcDNA4.1), PS1 WT or PS1 mutants were again transiently transfected into 2EB2 cells. Forty-eight hours after transfection, both the conditional medium and the cells were collected. ELISA was used to measure the levels of Aβ42 and Aβ40 in the conditional medium. Cells were lysed and the small peptides (tripeptide and tetrapeptide) were measured using LC-MS/MS (Agilent 6460, USA). ELISA results showed that compared to PS1 WT, the dominant negative mutation (D385A) did not affect the expression levels of Aβ42 and Aβ40, while most FAD PS1 mutants showed an increased Aβ42/Aβ40 ratio through different lines. For example, L166P increased the ratio of Aβ42/Aβ40 by lowering the level of Aβ40. I143V, M146V, G217A, E280A, L381V, and L392V increased the Aβ42/Aβ40 ratio through increased expression of Aβ42. The rest of the mutations increased the ratio of Aβ42/Aβ40 through both decreasing Aβ40 and increasing Aβ42 levels at the same time (**Figures 2A,B**). The amounts of tripeptide and tetrapeptide produced during the stepwise processing of longer form Aβ in living cells were then measured (**Figures 3** and **4**). The result indicated that neither the negative control nor any of the selected FAD PS1 mutations affected the selectivity of the γ-secretase cleavage of long form Aβ, judged by the total level of Aβ49-40 relative to that of total Aβ-related small peptides (**Figure 4A**). Compared to the PS1-WT, except for L166P and D385A mutations, most PS1 mutations were able to reduce the relative rate of Aβ42 cleavage into Aβ38. This reduction was concluded based upon the level of VVIA relative to that of total Aβ48-42 related small peptides (**Figure 4B**). Correspondingly, compared with PS1-WT, most FAD PS1 mutations (I143T, H163P, L166P, S170F, Q223R, M233V, E280A, and G384A) were able to decrease the relative rate of Aβ43 cleavage into Aβ40. This finding is based upon the level of IAT relative to that of the total Aβ49-40 related small peptides (**Figure 4C**). The LC-MS/MS results are consistent with the results from ELISA and Western Blot.

### Human FAD PS1 Mutations Differ in Their Effect on Aβ Generated by β-CTF Line

**FIGURE 5:**
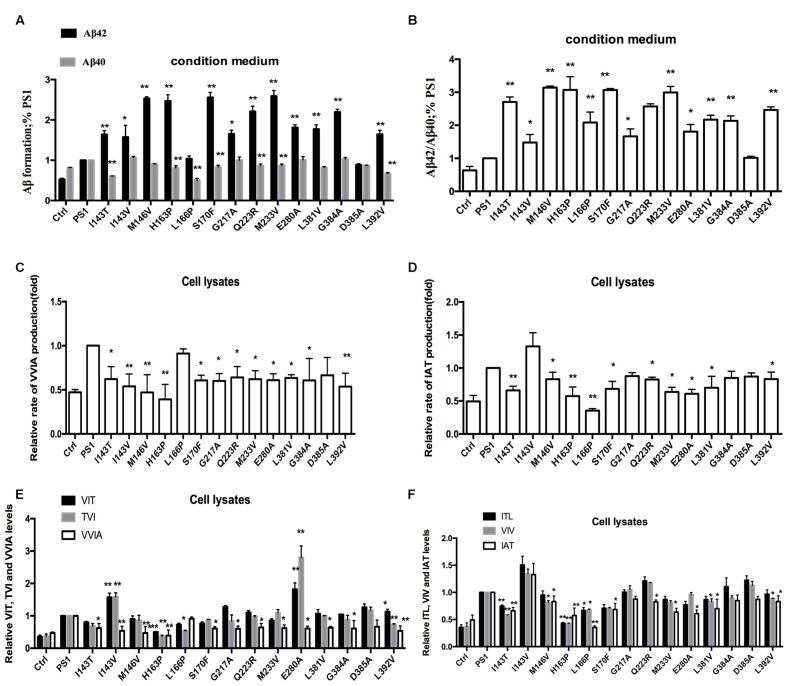
**Effects of PS1-WT and PS1 Mutant on CTF99 Cleavage. (A,B)** Conditioned medium from HEK293 co-expressing pzC99-flag and PS1-WT or FAD PS1 mutations were analyzed for levels of secreted Aβ40 and Aβ42 species using ELISA kit. **(A)** The levels of Aβ40 and Aβ42 are plotted in relative to PS1 WT. **(B)** The Aβ42/Aβ40 ratio. **(C)** Fold changes of the relative VVIA levels in cell lysates of HEK293 transfected pzC99 and PS1/PS1 FAD mutations. **(D)** Fold changes of the relative IAT levels in cell lysates of HEK293 transfected pzC99 and PS1/PS1 FAD mutations. **(E)** Fold changes of the relative VIT, TVI, and VVIA levels in cell lysates of HEK293 transfected pzC99 and PS1/PS1 FAD mutations. **(F)** Fold changes of the relative ITL, VIV, and IAT levels in cell lysates of HEK293 transfected pzC99 and PS1/PS1 FAD mutations. ^∗^*P* < 0.05; ^∗∗^*P* < 0.01.

Amyloid precursor protein can be cleaved into α-CTF (CTF83) and β-CTF (CTF99) by α-secretase and β-secretase *in vivo*, respectively. Therefore, we want to examine whether CTF83 can affect the CTF99 cleavage with PS1 mutations. pzC99, which is the cDNA encoding CTF99, was inserted into the pcDNA4.1 vector. We co-transfected transiently pzC99 with the empty vector, PS1 WT or PS1 mutants, into HEK293 cells. Forty-eight hours after transfection, both the condition medium and the cells were collected. The levels of Aβ42 and Aβ40 in the culture medium were similar to the previous ELISA results (**Figures 2A,B**). Most FAD PS1 mutations also increased the ratio of Aβ42/Aβ40 as shown before: L166P increased the ratio of Aβ42/Aβ40 by reducing Aβ40 level; I143V, M146V, G217A, E280A, L381V, and G384 increased the ratio of Aβ42/Aβ40 by increasing Aβ42 level; and finally, the rest of the mutations increased the ratio of Aβ42/Aβ40 by both reducing Aβ40 and increasing Aβ42 levels (**Figures 5A,B**). Cells were then lysed and the tripeptide and tetrapeptide were measured using LC-MS/MS (**Figure 3**). The results were in agreement with previous results in the Aβ48–Aβ38 line (**Figure 4D**). Compared with PS1-WT, some FAD PS1 mutations (I143T, I143V, M146V, H163P, S170F, G217A, Q223R, M233V, E280A, L381V, G384A, and L392V), showed elevated levels of Aβ42 as a result of lower VVIA, which was produced by the cleavage of Aβ42 into Aβ38, L166P and negative control had no effect on the VVIA level (**Figure 5C**). The results of Aβ49–Aβ40 line were slightly different (**Figure 4E**). Some FAD PS1 mutations (I143T, M146V, H163P, L166P, S170F, Q223R, M233V, E280A, L381V, and L392V) secreted lower levels of Aβ40, causing a lower level of IAT to be generated by the cleavage of Aβ43 into Aβ40. Others, such as I143V, G217A, and G384A, as well as the negative control, did not affect the IAT levels (**Figure 5D**). Among them, M146V, G384A, L381V, and L392V showed slightly different results from the previous data (**Figures 4B,C**). This could be due to CTF83 competing with CTF99 for cleavage by γ-secretase.

### Human FAD PS1 Mutations Differ in Their Effect on Aβ by α-CTF Line

**FIGURE 6:**
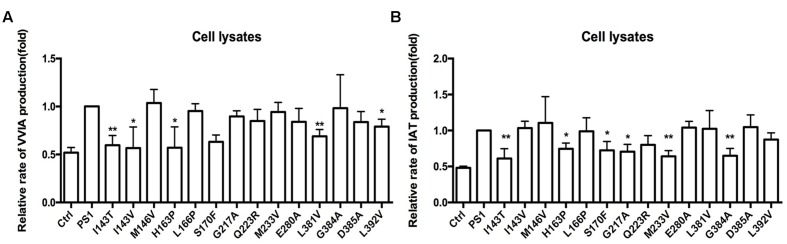
**Effects of PS1-WT and PS1 Mutants on CTF83 Cleavage. (A)** Fold changes of the relative VVIA levels in cell lysates of HEK293 transfected pzC83 and PS1-WT or FAD PS1 mutations. **(B)** Fold changes of the relative IAT levels in cell lysates of HEK293 transfected pzC83 and PS1-WT or FAD PS1 mutations. ^∗^*P* < 0.05; ^∗∗^*P* < 0.01.

The above results showed that FAD PS1 mutations differ in their effects on the Aβ generation line of APP and CTF99, especially in the Aβ49–Aβ40 line. APP could be cleaved by different secretase to generate α-CTF and β-CTF. Subsequently, they could be sequentially cleaved to produce Aβ fragments using γ-secretase. Both α-CTF and β-CTF can give rise to the tri- and tetra-peptides, but only β-CTF can eventually generate Aβ42. Thus, we further tested the hypothesis that the presence of CTF83 may affect the cleavage of CTF99. ELISA results showed that Aβ42 could not be detected in either PS1-WT or FAD PS1 mutations. The LC-MS/MS results showed that compared to PS-WT, some FAD PS1 mutations (I143T, I143V, H163P, S170F, L381V, and L392V) produced lower levels of VVIA (**Figure 6A**). Meanwhile, others (I143T, H163P, S170F, G217A, M233V, and G384A) produced lower levels of IAT (**Figure 6B**). Combined with the data of a specific peptide IAT cleaved from APP and a β-CTF cleavage using γ-secretase (**Figures 4** and **5**), we propose that the existenceof α-CTF may affect the β-CTF cleavage in certain, (i.e., M146V, L381V, G384A, and L392V) but not all, FAD PS1 mutations. Moreover, the presence of CTF83 may affect the cleavage of CTF99.

## Discussion

**FIGURE 7:**
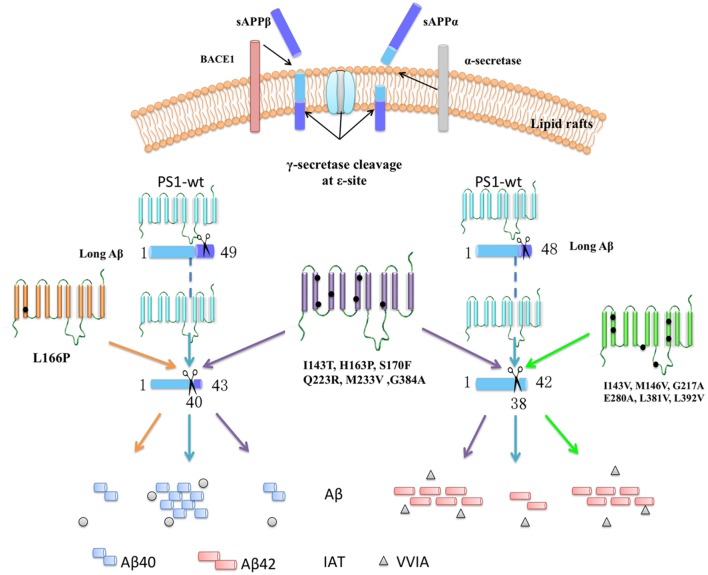
**Summarize the effects on specific peptides cleaved from long Aβ in different mutations.** The APP can be sequential cleavage by β-secretase (BACE1)/α-secretase and γ-secretase. APP-C-terminal fragment is cleaved at the 𝜀-site by γ-secretase, generating primarily long fragments, Aβ48 and Aβ49, followed by stepwise cleavage of every three amino acid residues at the C terminus, generating unique peptide. PS1 negative control, D385A, showed no change in unique peptide VVIA and IAT. Six mutations (I143T, H163P, S170F, Q223R, M233V, and G384A) change the ratio of Aβ42/40 by decreasing the cleavage of Aβ43-40 and Aβ42-38. L166P affect the ratio of Aβ42/40 by decreasing the cleavage of Aβ43-40. Six mutations (I143V, M146V, G217A, E280A, L381V, and L392V) affect the ratio of Aβ42/40 by decreasing the cleavage of Aβ42-38.

The PS1 mutation is known to be a key heredity factor of FAD. Mutation in PS1 can cause changes to γ-secretase activities, resulting in the alteration of the Aβ42/40 ratio. Recent research data show that Aβ can be generated by a series and continuous cleavage using γ-secretase (Takami et al., 2009; Okochi et al., 2013; Olsson et al., 2014). LC-MS/MS can be used to monitor the cleavage progress by following the tripeptides and tetrapeptides generated during the process. Based on the Aβ48–Aβ38 and Aβ49–Aβ40 cleavage lines, 14 PS1 mutations impacting the Aβ42/40 ratio via different lines of Aβ generated line were reported. Here, we discovered four significant findings. First, the PS1 negative control (D385A) showed no change in Aβ42, Aβ40 level, or Aβ42/40 ratio. This outcome is likely because they do not affect the cleavage of CTF83 and CTF99, especially the unique peptide VVIA and IAT. LC-MS/MS results indicated that they do not significantly affect the CTF83 and CTF99 cleavage line either. Second, six mutations (I143T, H163P, S170F, Q223R, M233V, and G384A) change the ratio of Aβ42/40 by decreasing the level of Aβ40 and increasing the level of Aβ42, respectively. Meanwhile, the cleavage of Aβ42 to Aβ38 and of Aβ43 to Aβ40 were both decreased. Such results indicate that those mutations affect the Aβ42/40 ratio through both the Aβ49-40 line and the Aβ48-42 line. Third, one mutation (L166P) affected the ratio of Aβ42/40 by decreasing the level of Aβ40 as a result of the decreased specific cleavage of Aβ43 to Aβ40, with no effect on Aβ42. This outcome indicates that L166P affects the Aβ42/40 ratio mainly through the Aβ49-40 line. Fourth, six mutations (I143V, M146V, G217A, E280A, L381V, and L392V) affected the ratio of Aβ42/40 by increasing the level of Aβ42, via specifically decreasing the cleavage of Aβ42 to Aβ38, with no effect on Aβ40. This outcome suggests that those mutations affect the Aβ42/40 ratio mainly through the Aβ48–Aβ40 line. To summarize, except for the negative control (D385A), which showed no effect on the Aβ42/40 ratio and the process of β-CTF cleavage, most of PS1 mutations could change the Aβ42/40 ratio through different long form Aβ cleavage lines (**Figure 7**).

Exactly how FAD mutations lead to the AD neuropathogenesis is still a mystery at present. One prevailing hypothesis is that PS mutations in mammalian systems cause the increase of the γ-secretase activity and enhance Aβ42 production (Shioi et al., 2007). Conversely, some new studies suggest that some mutations in FAD PS1 mutations (i.e., V82L, C263R et al.) not only would not increase production of Aβ42, but also lead to a loss of its essential functions. Many studies showed that FAD PS1 mutations would increase the ratio of Aβ42/Aβ40. However, previous studies primarily focus on, the lowered production of Aβ40, rather than the increased production of Aβ42 (Shen and Kelleher, 2007). Our data seems to be in agreeing with the previous hypotheses. Here, we show that L166p impacts the Aβ42/Aβ40 ratio by decreasing the level of Aβ40, rather than increasing the level of Aβ42 corresponds with previous work. Three PS1 mutations (L133P, G183V, and insR352) were found to cause a lack of amyloid pathology (Raux et al., 2000; Amtul et al., 2002; Dermaut et al., 2004) and an absence of Aβ accumulation. Such mutations are believed to be associated with Frontotemporal Dementia (FTD) more than with FAD, despite the fact they can be found in both FAD and FTD patients. The data we collected leads us to believe that most FAD PS1 mutations lead to amyloid pathogenesis. This outcome maybe due to FAD PS1 mutations function in regulating the cleavage of Aβ42–Aβ38 and Aβ43–Aβ40.

Interestingly, our results indicated that PS1 mutations have different effects on the β-CTF and APP processing of Aβ generation. It is known that APP can be cleaved by β-secretase and α-secretase, generating β-CTF and α-CTF. Subsequently, α-CTF and β-CTF may both be cleaved by γ-secretase. Additionally, α-CTF may remain the same peptide through the processing of γ-secretase cleavage. Here, we detected tri-peptides and tetra-peptides in the HEK293 cell linings co-transfected with the PS1 mutation and CTF83. We found that α-CTF is also processed by a series of continuous cleavages that produce the same tri-peptides and tetra-peptides as that of β-CTF. Moreover, the PS1 mutation could alter the cleavage process of α-CTF as well. Some mutations have different effects on the processing of α-CTF and β-CTF cleavages, suggesting that changes in the PS1 structure may decrease a β-CTF cleavage with no effect on α-CTF.

## Conclusion

Most mutations in PS1 accelerate the amyloid formation by affecting the Aβ generation process that results in a change of Aβ42/40 in FAD causing dementia.

## Author Contributions

NL and YQ contributed to the cell culture and sample preparation. ZR contributed to the data analysis. KL contributed to the sample detection by LC-MS/MS. DR and YD contributed to the experimental design and discussion. NL and HQ contributed to study design and manuscript preparation.

## Conflict of Interest Statement

The authors declare that the research was conducted in the absence of any commercial or financial relationships that could be construed as a potential conflict of interest.

## Abbreviations

Aβ: Amyloid β-peptide
APP: amyloid precursor protein
CTF: carboxy-terminal fragments
FAD: Familial Alzheimer’s disease
PS1: Presenilin1
TMD: transmembrane domains
