# Supplementary material for: Effect of Presenilin Mutations on APP Cleavage; Insights into the Pathogenesis of FAD
Source: Front Aging Neurosci. 2016 Mar 11;8:51. doi: 10.3389/fnagi.2016.00051 (PMC4786568; doi:10.3389/fnagi.2016.00051)
Supplement: Supplementary file 1 [file Table_1.DOCX]

Table.S1 the primers of overlap extension PCR

| PS1 Rv | 5’ GGCGAATTCGTTCTAGATATAAAATTGATGGA 3’ |
| --- | --- |
| PS1 Fw | 5’CCCAAGCTTAAATGACAGAGTTACCTGC 3’ |
| I143T Rv | 5’ GGAGGATAGTCAQTGACAACAGTGACACTGATCATG 3’ |
| I143T Fw | 5’CATGATCAGTGTCACTGTTGTCATGACTATCCTCC 3’ |
| I143V Rv | 5’ GGAGGATAGTCATGACAACAACGACACTGATC 3’ |
| I143V Fw | 5’ GATCAGTGTCGTTGTTGTCATGACTATCCTCC 3’ |
| M146V Rv | 5’ CCAGGAGGATAGTCACGACAACAATGCAGGC 3’ |
| M146V Fw | 5’ GCCTGCATTGTTGTCGTGACTATCCTCCTGG 3’ |
| H163P Rv | 5’ATAATAAGCCAGGCAGGGATGACCTTATAGCACC 3’ |
| H163P Fw | 5’ GGTGCTATAAGGTCATCCCTGCCTGGCTTATTAT 3’ |
| L166P Fw | 5’ GCCTGGCCTATTATATCATCTCTATTGTTGCTGTTC |
| L166P Rv | 5’ GAACAGCAACAATAGAGATGATATAATAGGCCAGGC |
| S170F Rv | 5’ GCAACAATAGAAATGATATAATAAGCCAGGC 3’ |
| S170F Fw | 5’ GCCTGGCTTATTATATCATTTCTATTGTTGC 3’ |
| Q223R Rv | 5’ CATAATGAGATATGCCCGCTGGAGTCGAAGT 3’ |
| Q223R Fw | 5’ ACTTCGACTCCAGCGGGCATATCTCATTATG 3’ |
| M233V Rv | 5’ATGATTAGTGCCCTCGTGGCCCTGGTGTTTATC 3’ |
| M233V Fw | 5’ GATAAACACCAGGGCCACGAGGGCACTAATCAT 3’ |
| E280A Rv | 5’ CTGGAAAAAGCGTTGCATTTCTCTCCTGAGCTGT 3’ |
| E280A Fw | 5’ ACA GCTCAGGAGAGAAATGCAACGCTTTTTCCAG 3 |
| L381V Rv | 5’ ATCTCCCAATCCAACTTTTACTCCCCT 3’ |
| L381V Fw | 5’AGGGGAGTAAAAGTTGGATTGGGAGAT 3’ |
| G384A Rv | 5’ CACTGTAGAAAATGAAATCTGCCAATCCAAG 3’ |
| G384A Fw | 5’ CTTGGATTGGCAGATTTCATTTTCTACAGTG 3’ |
| D385A Rv | 5’ CACTGTAGAAAATGAAAGCTCCCAAGCCAAG 3’ |
| D385A Fw | 5’ CTTGGCTTGGGAGCTTTCATTTTCTACAGTG 3’ |
| L392V Rv | 5’ GCTGAGGCTTTACCAACCACAACACTGTAGA 3’ |
| L392V Fw | 5’TCTACAGTGTTGTGGTTGGTAAAGCCTCAGC 3’ |
